# The Impact of Human Papillomavirus (HPV) Associated Oropharyngeal Squamous Cell Carcinoma (OPSCC) on Nutritional Outcomes

**DOI:** 10.3390/nu13020514

**Published:** 2021-02-04

**Authors:** Jane Harrowfield, Elizabeth Isenring, Nicole Kiss, Erin Laing, Ruby Lipson-Smith, Ben Britton

**Affiliations:** 1 Nutrition and Speech Pathology Department, Peter MacCallum Cancer Centre, Melbourne, 3000, Australia; Jane.Harrrowfield@petermac.org (J.H.); Erin.Laing@petermac.org (E.L.); 2 Faculty of Health Sciences and Medicine, Bond University, Gold Coast 4226, Australia; Lisenrin@bond.edu.au; 3 Institute for Physical Activity and Nutrition, Deakin University, Geelong 3220, Australia; Nicole.Kiss@deakin.edu.au; 4 Allied Health Research, Peter MacCallum Cancer Centre, Melbourne 3000, Australia; 5 Department of Health Services Research, Peter MacCallum Cancer Centre, Melbourne 3000, Australia; ruby.lipson-smith@florey.edu.au; 6 The Florey Institute of Neuroscience and Mental Health, Heidelberg 3084, Australia; 7 Hunter New England Health, Newcastle 2305, Australia; 8 School of Medicine and Public Health, University of Newcastle, Newcastle 2308, Australia; 9 Hunter Cancer Research Alliance, Newcastle 2305, Australia

**Keywords:** oropharyngeal cancer, OPSCC, head and neck, human papillomavirus, HPV, malnutrition, weight loss, nutrition, PG-SGA

## Abstract

Background: Patients undergoing (chemo) radiotherapy for oropharyngeal squamous cell carcinoma (OPSCC) are at high risk of malnutrition during and after treatment. Malnutrition can lead to poor tolerance to treatment, treatment interruptions, poor quality of life (QOL) and potentially reduced survival rate. Human papillomavirus (HPV) is now known as the major cause of OPSCC. However, research regarding its effect on nutritional outcomes is limited. The aim of this study was to examine the relationship between HPV status and nutritional outcomes, including malnutrition and weight loss during and after patients’ (chemo) radiotherapy treatment for OPSCC. **Methods:** This was a longitudinal cohort study comparing the nutritional outcomes of HPV-positive and negative OPSCC patients undergoing (chemo) radiotherapy. The primary outcome was nutritional status as measured using the Patient Generated-Subjective Global Assessment (PG-SGA). Secondary outcomes included loss of weight, depression, QOL and adverse events. **Results:** Although HPV-positive were less likely to be malnourished according to PG-SGA at the beginning of treatment, we found that the difference between malnutrition rates in response to treatment was not significantly different over the course of radiotherapy and 3 months post treatment. HPV-positive participants had significantly higher odds of experiencing >10% weight loss at three months post-treatment than HPV-negative participants (OR = 49.68, 95% CI (2.7, 912.86) *p* ≤ 0.01). **Conclusions:** The nutritional status of HPV positive and negative patients were both negatively affected by treatment and require similarly intense nutritional intervention. In acute recovery, HPV positive patients may require more intense intervention. At 3- months post treatment, both groups still showed nutritional symptoms that require nutritional intervention so ongoing nutritional support is essential.

## 1. Introduction

The prevalence of malnutrition in people with head and neck cancer (HNC) is one of the highest of all cancers [1,2]. People with oropharyngeal squamous cell carcinoma (OPSCC) are particularly susceptible as the specific tumor site makes dysphagia and odynophagia common [3,4]. In addition, treatment for OPSCC often includes radiotherapy (RT) combined with chemotherapy. This can result in significant acute toxicity including mucositis, dysphagia, anorexia, dysgeusia, fatigue leading and longer-term effects such as radiotherapy induced osteoradionecrosis which can lead to malnutrition, both long and short term. Malnutrition can lead to poor tolerance to treatment, treatment interruptions, poor quality of life (QOL), need for feeding tube (FT) insertion, long term gastrostomy tube dependence and potentially reduced survival rate in people with OPSCC who are undergoing treatment [1,2,3,5]. These morbidities worsen with increasing treatment intensity and with certain types of chemotherapy such as cisplatin which is recommended for OPSCC treatment [6,7].

The prevalence of OPSCC is increasing in higher socioeconomic countries despite the relatively stable incidence of other HNC [8]. This is due to the increase in human papillomavirus (HPV)-associated OPSCC which now accounts for approximately two thirds of all OPSCC in countries such as Australia [9]. HPV is a sexually transmitted disease of which 15 types have been associated cancer. HPV 16 and 18 are particularly associated with mucosal cancers and HPV 16 accounts for up to 90% of all HPV associated HNC [8]. HPV is often diagnosed with histopathology using p16 as a surrogate marker for presence of HPV though newer evidence recommends bimodal diagnosis with the addition of DNA- and mRNA-based polymerase chain reaction (PCR) and in situ hybridization (ISH) [10]. People with HPV-positive OPSCC have a better five-year survival rate compared to people with HPV-negative OPSCC and are often younger and less likely to have a smoking and drinking history and more likely to have had a greater number of sexual partners [11]. People with HPV-positive OPSCC often present without pre-existing symptoms that affect nutritional intake such as pain and dysphagia and therefore may be less likely to be malnourished at commencement of treatment than HPV-negative OPSCC patients [12]. The relationship between HPV-positive status in OPSCC patients and malnutrition are not well studied.

Patients with HPV-positive OPSCC are known to have better nutritional status at diagnosis than people with HPV-negative OPSCC [13,14,15]. Studies comparing the prevalence of malnutrition in HPV-positive and negative patients after treatment report similar, if not worse, nutritional outcomes for people who are HPV-positive compared to those who are HPV-negative [13,14]. This suggests that people who are HPV-positive are susceptible to a greater decline in nutritional status and potentially at greater risk of treatment-related malnutrition than people who are HPV-negative. Some studies attribute the comparatively worse nutritional outcomes in HPV-positive patients to lack of adherence to nutritional guidelines which leads to delayed feeding tube insertion and initiation of feeding [13,15]. Other studies suggest that patients with HPV-positive status are at greater risk of a decline in nutritional status due to experiencing worse treatment toxicities [14]. Treatment de-escalation, defined as a reduction or alteration of chemotherapy and/or radiotherapy in order to minimize treatment burden in HPV-positive patients, has been proposed with some promising results in nutritional and swallowing outcomes [16]. However, a recent, large, randomized, controlled trial (RCT) found that chemotherapy de-escalation resulted in reduced survival, increased locoregional recurrence and had no advantage in long term toxicity [7]. As a result, treatment pathways remain the same for HPV-positive and negative patients.

While research is emerging regarding the impact of HPV status on malnutrition, there are several gaps including a lack of focus on patients specifically with OPSCC and inconsistencies in regard to measurement tools and data collection time points. Early studies often compared HPV-positive and negative patients in a heterogeneous HNC population but we now know that the causal link between HPV and survival is unique to OPSCC [17]. Recent research regarding the prevalence of malnutrition is scarce and has only included percentage weight loss or categorical reference ranges which vary (e.g., ≥5% or ≥10% weight loss) instead of a more comprehensive and validated tool to diagnose malnutrition [13,14]. It is now well accepted that weight loss alone is insufficient to diagnose malnutrition in cancer patients and, as a result, more specific diagnostic tools have been validated in this population [18,19]. Finally, most HPV comparison studies do not take into account the differences in baseline malnutrition between HPV positive and negative patients, confounding the inferences that can be made about the differential effects of radiotherapy on HPV-positive patients. There is also a lack of research examining nutritional outcomes post treatment and at multiple time points. This is important as poor nutritional status can continue far past cessation of treatment [20,21,22]. Factors such as depression and poor QOL are also known to effect nutritional outcomes in the wider HNC population [23,24] but their relationship with HPV status and nutritional outcomes has not been well studied. Therefore, these factors were assessed in our study.

Despite the better prognosis associated with HPV-positive OPSCC, treatment remains the same and the risk of poor nutritional outcomes is high. A better understanding of nutritional outcomes in relation to HPV status could help to inform targeted nutritional interventions and therefore contribute to better nutritional and treatment outcomes for people who are undergoing treatment for HPV-positive OPSCC. The aim of this study was to examine the relationship between HPV status and nutritional outcomes, including malnutrition and weight loss during and after patients’ (chemo) radiotherapy treatment for OPSCC. Based on previous research, we hypothesized that patients with HPV-positive status would experience more malnutrition (primary outcome), more weight loss, more depression, lower QOL and more adverse events (secondary outcomes) during and after treatment compared to patients with HPV-negative status.

## 2. Materials and Methods

### 2.1. Study Design

This longitudinal, cohort study was a sub-study of a stepped wedge, cluster, randomized controlled trial titled, ‘Eating As Treatment (EAT)’. Detailed information on the original study protocol is published elsewhere [25]. Data collected during EAT from two sites, Peter MacCallum Cancer Centre, Melbourne and Princess Alexandra Hospital, Brisbane were used for this sub-study, as p16 status is routinely collected at these sites as a marker of HPV status.

### 2.2. Participants

Eligible participants were selected from the two participating sites. Informed, written consent was provided in the original trial for data to be used for related studies. EAT was approved by Hunter New England Health (HREC/12/HNE/108).

All outcome assessments were conducted by an independent researcher at four time points: first week of radiotherapy (RT), last week of RT, one-month post-RT and three-months post-RT. All baseline characteristics and covariates such as tumor stage and treatment type were collected by a research assistant via chart review with the exception of p16 status which was retrospectively collected from confirmed histopathology reports.

### 2.3. Inclusion Criteria

Eligible patients were aged 18 years or older who had pathologically confirmed diagnosis of OPSCC. Immunohistochemistry (IHC) staining using Roche CINtec histology kit, clone E6H4was used to confirm p16 status. They were planned for definitive or postoperative radiotherapy with curative intent (chemoradiation permitted including neoadjuvant and adjuvant chemotherapy), the prescribed radiation dose being at least 60 gray as regional nodal irradiation. They were available for follow-up for at least 6 months post study initiation and provided written, informed consent. Patients were excluded if they were unable to communicate in English; had organic brain disease (impairing ability to complete questionnaires), had T1 or T2 glottic carcinoma undergoing small fields radiation therapy or T1 and T2 tonsil cancer undergoing unilateral treatment.

### 2.4. Primary Outcome

The primary outcome of nutritional status was measured using the Patient Generated -Subjective Global Assessment (PG-SGA) [26]. The PG-SGA is reliable and validated in an oncology population and in Australia, is the recommended assessment tool for malnutrition in the oncology population [18,27]. It produces a score between 1–49 (where 1 requires no intervention, 2–3 requires low intensity intervention, 4–8 indicates dietetic intervention dependent on symptoms and ≥9 indicates critical need for dietetic intervention) and a categorical global assessment (A-indicating well nourished, B-indicating moderately malnourished or C-indicating severely malnourished). The score incorporates prognostic factors of nutritional status such as weight change, dietary intake, factors affecting nutritional intake, metabolic stress, subcutaneous fat and muscle wastage and presence of disease.

### 2.5. Secondary Outcomes

Secondary outcome measures included loss of weight (LOW), depression, QOL and adverse events including treatment interruptions, unplanned hospital admissions and length of stay (LOS) of these admissions, reactive nasogastric tube (NGT) insertions for feeding, dietary adequacy, nutrition impact symptoms (NIS) and mortality at two years post-treatment.

### 2.6. Loss of Weight

The weight (kg) of participants was recorded using electronic standing scales, at all-time points. Percentage LOW from baseline was calculated at each time point. LOW was also categorized into >5% LOW and >10% LOW as these are cut off points used in other literature for important weight loss and used clinically to indicate significant weight loss. 

### 2.7. Depression

Depression was assessed using the validated Patient Health Questionnaire 9 (PHQ-9) [28]. This is a self -administered tool to measure the severity of 9 diagnostic criteria for depression. It provides a score of depression severity 0–27 (27 as the highest severity) with a score of ≥14 indicating the presence of major depressive disorder.

### 2.8. Quality of Life

QOL was assessed using the European Organization for Research and Treatment of Cancer, Core Quality of Life Questionnaire (EORTC QLQ-C30). This is a 30-item self-report questionnaire designed and validated to measure QOL in patients with cancer [29]. It includes 5 functional scales (physical, role, cognitive, emotional and social), 3 symptom scales (fatigue, pain, nausea and vomiting), a global health status scales and 6 single items assessing the financial impact of disease and commonly reported symptoms (dyspnea, loss of appetite, insomnia, constipation and diarrhea).

### 2.9. Dietary Adequacy, NIS and Reactive NGT Insertions

Dietary adequacy was obtained from a section (Box 2) of the PG-SGA. Scores for Box 2 are measured on a scale of 0–5 (0 indicating normal nutritional intake and 5 indicating eating very little of anything). NIS were obtained from Box 3 of the PG-SGA. Scores for Box 3 are measured on a scale of 0–24 (0 indicating the patient has no symptoms that are affecting intake and 24 indicating that intake is severely impacted by symptoms). Reactive NGT insertions were measured as the proportion of patients with NGT inserted after the start of treatment in relation to poor oral intake.

### 2.10. Adverse Events

Treatment interruptions were measured as total number of participants with unplanned interruptions to their radiotherapy schedule. Unplanned hospital admissions were measured as total number of admissions from start of radiotherapy to 3 months post treatment and LOS was calculated as a total of all participants’ hospital stays for each HPV status group. Mortality was measured as proportion of participants who had died by two-years post treatment.

### 2.11. Sample Size

This exploratory study used an opportunistic sample of patients with OPSCC who participated in a large multi-center trial, examining the entirety of OPSCC patients from two of the sites. 

All analyses were conducted using STATA 13 [30]. The primary outcome, PG-SGA score, was analyzed using a Linear Mixed Models (LMM) regression. The assumptions of LMM were assessed by inspecting appropriate residual plots. The model used an unstructured covariance examining differences between HPV-positive and negative patients and controlled for the effects of the EAT trial intervention, differences between hospitals, time of assessment (start of RT, end of RT, 1-month post-RT, 3-months RT), calendar date, tumor site, tumor stage and baseline nutritional status. The model included a random individual level intercept to account for the repeated measures on individuals over assessment time-points.

Secondary outcomes were assessed using the same model to analyze the differences across all time points. Percentage LOW, depression (PHQ-9) and QOL (QLQ C30) were analyzed using LMM while the categorical variables such as the PG-SGA, >5 and >10% weight loss were analyzed using logistic mixed effects models. 

Treatment interruption was analyzed using a logistic regression. Unplanned hospital admissions were analyzed using a negative binomial regression. Both models included HPV status, intervention group, baseline nutritional status, hospital and calendar time.

## 3. Results

### 3.1. Participants

Data was available from 83 participants (70 HPV-positive and 13 HPV-negative) from the two sites between July 2013 and January 2016. There was one dropout from each of the p16 positive and negative groups (*n* = 2). Both of these were lost to follow-up post-treatment, the reasons stated were; living in rural areas and not able to return to hospital for follow up assessments. None of the participants died during the course of the study. Patient characteristics at baseline can be seen in Table 1.

### 3.2. Primary Outcome

Malnutrition

**Figure 1:**
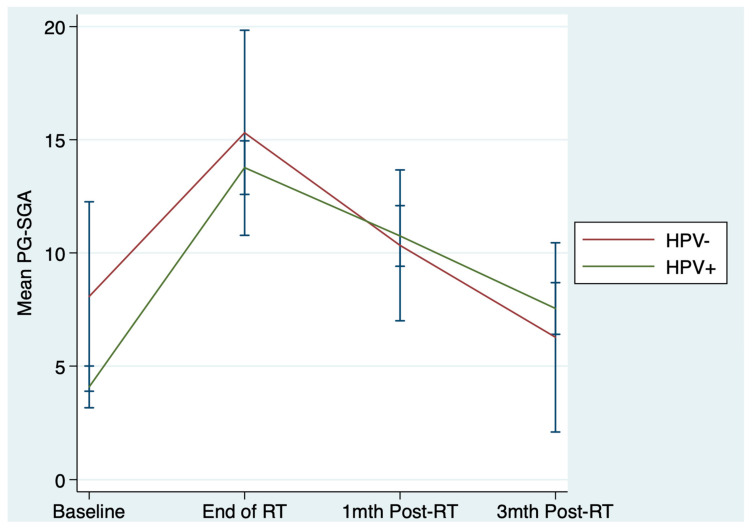
Mean PG-SGA (Patient Generated-Subjective Global Assessment) score by HPV (Human papillomavirus) status over all timepoints with 95% Confidence intervals.

Mixed models controlling for variables such as baseline nutritional status and the effect of the larger trial intervention (see statistical methods above), found no significant difference in PG-SGA category and scores between HPV-positive and negative patients over the course of treatment and recovery (Table 2 and Figure 1).

### 3.3. Secondary Outcomes

#### 3.3.1. Loss of Weight

HPV positive participants had significantly higher odds of experiencing >10% weight loss at three-months post-treatment than HPV negative participants (OR = 49.68, 95%CI (2.7, 912.86) *p* ≤ 0.01) (Table 3). The average difference between groups across all time points was not significant.

#### 3.3.2. Depression

Across all time points, there were no statistically significant differences in depression scores between HPV-positive and HPV-negative patients (Table 3).

#### 3.3.3. Adverse Events

HPV-positive patients had statistically significant higher odds of unplanned admissions than HPV-negative patients (OR = 3.00, 95% CI (1.12, 8.02), *p* = 0.03) (Table 3). Mortality at 2 years post treatment was 1.5 times greater for HPV-positive patients compared to HPV-negative patients (30% versus 7%, *p* < 0.01).

#### 3.3.4. Reactive NGT Insertions, Dietary Adequacy and Nutrition Impact Symptoms

There was no statistically significant difference in reactive NGT insertions, dietary adequacy and NIS between groups (Table 3).

#### 3.3.5. Quality of Life

There was no statistically significant difference in global QOL scores, symptom and functional scale scores between groups across all time points (Table 4). The exception is cognitive decline which was worse for HPV positive patients (*p* < 0.01).

## 4. Discussion

This is the first longitudinal study, to our knowledge, in which the impact of HPV status on malnutrition and related factors has been investigated at various time points during and after RT treatment using a diagnostic tool validated in the oncology population. The detrimental effects of treatment in the heterogenous HNC population has been widely studied and, as a result, nutritional guidelines have been established [27]. These guidelines are based on the wider HNC population and are not specific to the HPV-positive patients who now account for the majority of OPSCC patients and where malnutrition at diagnosis is less prevalent. We found that treatment has a similarly detrimental effect on nutritional outcomes for HPV-positive as negative OPSCC patients and therefore these patients require equivocal if not more intense nutritional intervention.

In contrast to previous studies [13,14], we did not find worse nutritional outcomes for the HPV-positive group at cessation of treatment. These unexpected results were likely partly due to the relative worse nutritional outcomes for HPV-negative patients in our study compared to HPV-negative patients included in previous research. The difference in nutritional outcomes between studies could also be explained by differences in measurement of malnutrition, as these previous studies used weight loss alone as a marker of nutritional status whereas the more comprehensive PG-SGA was used in this current study. Weight loss alone could potentially underestimate malnutrition for HPV-negative patients.

The PG-SGA is the recommended tool for malnutrition assessment in radiotherapy patients in Australia [27]. It has shown to have high specificity and reliability in the oncology population [18]. This tool was utilized as it includes a description of symptoms commonly affecting the nutritional status of oncology patients, which can then be addressed by nutritional intervention. Other single measure tools such as biochemical and anthropometrical markers have inherent difficulties with interpretation and measurement and do not allow for the underlying symptoms to be identified and addressed.

Differences in treatment modality may be an additional explanation for the difference between the results of our study and previous studies. Concurrent chemotherapy administration and intensity of RT are known to worsen nutritional outcomes [31,32]. While in our study HPV-positive and negative patients had similar rates of adjuvant chemotherapy and RT doses, HPV-negative patients in previous studies had less intense treatment [13,14]. This could increase the difference between HPV-positive and negative patients in previous work. It is also acknowledged that in our study, while the number of ≥3 stage disease included in the study is similar, there were more Stage 4 OPSCC patients who could have greater nutritional symptoms in the HPV negative group. This could decrease the difference between groups in our study given the potential for worse nutrition related symptoms with higher stage disease.

We did find evidence of more severe weight loss at 3 months post treatment in HPV-positive OPSCC patients suggesting a slower recovery for these patients. This more severe weight loss was unlikely due to worse toxicities as patient-reported symptoms were similar for HPV-positive and negative patients overall and unplanned admissions, an indirect measure of treatment toxicity, were predominately seen during treatment. HPV-positive patients did have significantly higher BMI at the beginning and end of treatment which may have lessened both patients’ and clinicians’ concern regarding weight loss and reduced likelihood of adherence to nutritional guidelines. Future research is needed to confirm this potential slower recovery post treatment in HPV-positive patients and the reasons for this so that the appropriateness of current recommendations for nutritional and supportive care post-treatment for HPV-positive patients can be investigated. This is especially important given that the frequency of follow up recommended for HNC patients is based on patients undergoing radiotherapy alone and current best practice treatment for this patient group has the added detrimental impact on nutrition associated with chemotherapy treatment [27]. Given the known increased 5-year survival rate for these patients, longer term research beyond 3 months post treatment is also warranted.

Previous studies [13,15] suggest lack of adherence to guidelines in relation to insertion and timing of insertion of feeding tubes (FT) in the HPV-positive population contributes to poorer nutritional outcomes. Therefore, closer adherence to these guidelines for both HPV-positive and negative OPSCC in our study may help to explain the similar nutritional outcomes. FT insertion is generally recommended at 5% LOW for HNC patients undergoing treatment [33]. In our study, 87% of HPV-positive patients and 83% of HPV-negative patients had >5% LOW and 64% and 69% respectively had a feeding tube inserted. Vangelov et al. found slightly more FT insertions for HPV-positive than HPV-negative patients (64% vs. 60%). However, as ≥5% LOW was seen in 94% of HPV-positive patients and 60% of HPV-negative patients, the proportion of patients potentially requiring FTs and not receiving them is higher in the HPV-positive group compared to HPV-negative group. In our study, prophylactic feeding was slightly lower for HPV-positive patients (34%) compared to HPV-negative OPSCC patients (46%) while rates of reactive NGT insertion was no different. Vangelov et al. [11] found greater differences in prophylactic feeding rates between the two groups (24% for HPV-positive vs. 50% for HPV-negative) potentially leading to greater weight loss in the HPV-positive group. Current guidelines [27] recommend that prophylactic FT insertion be considered in HNC patients undergoing treatment. Predictors of patients who are most likely to require them are well studied and include poor nutritional status at presentation [22,34,35]. Results of our study show that favorable nutritional status at presentation does not prevent decline in the nutritional status of HPV-positive patients and so may not be useful in determining appropriateness for prophylactic feeding in this population. Predictive factors for HPV status and prophylactic FT insertion in HPV positive patients could be the subject of future research.

The significant difference in unplanned admissions is interesting. It may be an indirect marker for clinician-reported treatment toxicity given patients are usually admitted for management of side effects. This is supported by previous evidence that clinician-reported toxicities are worse for HPV-positive patients though this same study reported no difference in unplanned admissions [14]. Given we found no difference in patient reported toxicities, our results support these previous findings that treatment toxicity and unplanned admissions are not correlated in the HPV population [14]. The lack of RT interruptions seen in HPV-positive patients, albeit only a trend compared to HPV-negative patients, may have contributed to unplanned admissions indirectly as the quest to avoid interruptions can lead to admission for symptom management. Some research suggests patients who start treatment with less pre-existing symptoms predict better future QOL outcomes and this, in turn, relates to worse patient-reported outcomes in recovery [36,37]. Therefore, HPV-positive patients may be more likely to perceive functional outcomes as severe during and after treatment and therefore seek admission. Psychological interventions may be useful to address patients’ perceptions and adherence to nutritional guidelines. Successful interventions have been studied in the wider HNC population and have been shown to improve adherence to nutritional guidelines, nutritional outcomes and QOL [25,38]. There are currently no known psychological interventions in the HPV-positive population and more research in this population may be warranted.

The higher proportion of HPV-positive patients than HPV-negative patients in the OPSCC population is a limitation of this and earlier studies. The difference in sample size between groups could explain the lack of statistical significance found. This study was a post-hoc sub-study of a previous work which meant the sample is limited and a prospective sample size calculation was not conducted. The sample, while not small, was not balanced (*n* = 70 vs. *n* = 13). The study had 90% power to find a large effect (Cohen’s *d* = 0.8), 53% power to find a medium effect (*d* = 0.5) and only 12% power to find a small effect (*d* = 0.2). As such, non-significant findings should be viewed in the context of this sample not being powered to find smaller effect sizes and the subsequent higher risk of Type 2 error. Further research should address this limitation.

The current study was conducted in tertiary treatment centers with well-established multidisciplinary teams, so findings may not be generalizable to smaller treatment centers. There may have been individual differences in nutritional intervention as nutritional interventions were undertaken by different clinicians. As mentioned in the introduction, bimodal detection of HPV status is now recommended and this was not available in this study which could potentially overestimate positive HPV cases.

## 5. Conclusions

In this first study of longitudinal change of nutritional status in relation to HPV status in OPSCC using validated measures, two main outcomes are seen. Firstly, we found the impact of treatment on HPV-positive patients’ nutritional status, whose baseline nutrition was superior, was not significantly different by the final week of RT treatment to that of HPV-negative patients and this continued into the post-treatment period. Secondly, HPV-positive patients had significantly greater patients with >10% LOW at 3 months post treatment and significantly more unplanned admissions. We found similar rates of malnutrition were associated with similar % weight loss, depression scores, QOL and reactive NGT rates. Our results differ to other studies in that HPV-positive patients did not have worse nutritional outcomes by the end of treatment. This is likely due to difference in research methodology and potentially due to better adherence to guidelines for HPV-positive patients in our study. Nevertheless, the nutritional status of HPV positive and negative patients were both negatively affected by treatment and require similarly intense nutritional intervention during treatment. In acute recovery, HPV positive patients may require more intense intervention. At 3-months post treatment, both groups still showed nutritional symptoms that require nutritional intervention so ongoing nutritional support is essential. Further prospective research with a larger sample size is needed to confirm the results found in this study and could include sociological and psychological exploration with a focus on interventions to improve adherence to nutritional guidelines and support to help manage patient expectations. The superior survival outcomes for HPV-positive patients mean these patients are living longer so a study of long-term effects of treatment on nutritional status and contributing factors along with appropriate nutritional intervention is warranted.

## Figures and Tables

**Table 1:** Patient characteristics at baseline.

Variable		HPV-Negative	HPV-Positive
		*n* = 13 (16%)	*n* = 70 (84%)
Categorical variables, *n (%)*			
Male		12 (92)	61 (87)
Aboriginal or Torres Strait Islander		0	2 (3)
Non-English speaker at home		1 (8)	1 (1)
Marital status			
	Married/Defacto	9 (69)	44 (63)
	Separated/Divorced/Widowed	2 (15)	17 (24)
	Single/Never married	2 (15)	9 (13)
Highest level of education			
	Primary school	6 (46)	6 (9)
	High school	3 (23)	23 (33)
	University/Vocational college	4 (31)	41 (59)
Tumor stage (AJCC 7)			
	I	0	2 (3)
	II	2 (15)	10 (14)
	III	0	14 (20)
	IV	11 (85)	44 (63)
Concurrent chemotherapy		13 (100)	61 (87)
Post-operative radiotherapy		0 (0)	9 (13)
Prophylactic PEG		4 (31)	22 (31)
Prophylactic NGT		2 (15)	2 (3)
Received EAT intervention		7 (54)	42 (60)
Substance use			
Harmful Alcohol Use and Likely Dependence (AUDIT ≥8)		5 (38)	18 (26)
Reported current smoking		3 (23)	8 (11)
Nicotine Dependence (AUDIT ≥8)		6 (46)	3 (4)
Continuous variables, mean (s.d)			
Age in years		56 (9.4)	57 (7.4)
Prescribed radiation (Gy)		69 (2.8)	69 (8.3)
Depression (PHQ-9)		4.1 (4.1)	3.4 (3.7)
Baseline Nutrition Variables			
PG-SGA score mean (s.d)		8.1 (6.9)	4.1 (3.9) *
PG-SGA category (B/C) *n* (%) #		6 (46)	8 (12) *
BMI in Kg/m^2^ mean (s.d)		24.5 (5.3)	29.7 (6.2) *

Abbreviations; AJCC 7 = American Joint Committee on Cancer, Version 7; AUDIT ≥ 8 = Alcohol Use Disorders Identification Test; BMI = Body Mass Index; EAT = Eating As Treatment; Gy = gray; HPV = Human papillomavirus; NGT = Nasogastric Tube; PEG = Percutaneous Endoscopic Gastrostomy; PG-SGA = Patient Generated-Subjective Global Assessment; PHQ-9 = Patient Health Questionnaire 9; s.d = standard deviation. * Significant difference between groups (*p* < 0.01). # Category A = Well Nourished; B = Moderately malnourished; C = Severely malnourished.

**Table 2:** Primary outcome: HPV-positive and HPV-negative in a linear mixed model of mean PG-SGA score and category across end of treatment, 1 months and 3 months post treatment.

Variable		HPV-Negative *n* = 13	HPV-Positive *n* = 70	Statistic	*p*-Value	95% CI
PG-SGA score, mean (s.d) *				*β* = 0.80	0.44	−1.22, 2.82
	Last wk treatment	15.3 (7.5)	13.8 (4.9)			
	1-mth post-treatment	10.3 (5.2)	10.8 (5.5)			
	3-mth post-treatment	6.3 (6.2)	7.5 (4.7)			
PG-SGA category B/C, *n* (%) #				OR = 0.50	0.30	0.14, 1.83
	Last wk treatment	11 (85)	62 (89)			
	1-mth post-treatment	8 (62)	50 (71)			
	3-mth post-treatment	5 (38)	30 (43)			

Abbreviations; HPV = Human papillomavirus; CI = confidence interval; OR = odds ratio; PG-SGA = Patient Generated-Subjective Global Assessment; s.d = standard deviation. * Higher score indicates worse nutritional status or risk. # Category A = Well Nourished; B = Moderately malnourished; C = Severely malnourished.

**Table 3:** Secondary outcomes: HPV-positive and HPV-negative in a linear mixed model of mean LOW, depression, PG-SGA Box 2 and 3 across end of treatment, 1 months and 3 months post treatment.

Variable		HPV-Negative *n* = 13	HPV-Positive *n* = 70	Statistic	*p*-Value	95% CI
% LOW, mean (s.d)				β = −1.93	0.15	−4.51, 0.66
	Last wk treatment	8.5 (5.4)	7.1 (4.5)			
	1-mth post-treatment	11.2 (5.9)	10.2 (6.8)			
	3-mth post-treatment	9.9 (9.9)	12.8 (7.6)			
>5% LOW, *n* (%)						
	Last wk treatment	9 (69)	48 (69)	OR = 1.12	0.81	0.46, 2.70
	1-mth post-treatment	10 (77)	56 (80)			
	3-mth post-treatment	10 (77)	61 (87)			
>10% LOW, n (%)				OR = 0.68	0.29	0.05, 9.31
Last wk treatment		4 (31)	19 (27)			
1-mth post-treatment		7 (54)	37 (53)			
3-mth post-treatment		4 (31)	47(67)	OR = 49.68	<0.01	2.7, 912.86
Depression, mean (s.d)				β = 0.49	0.66	−1.72, 2.70
	First wk treatment	4.2 (4.1)	3.4 (3.7)			
	Last wk treatment	7.1 (3.2)	9.9 (5.5)			
	1-mth post-treatment	6.3 (5.1)	6.3 (4.9)			
	3-mth post-treatment	7.7 (6.5)	4.6 (4.9)			
PG-SGA Box 2 mean (s.d)				β = 0.33	0.14	−0.11, 0.78	
First wk treatment		0.9 (1.1)	0.4 (0.8)			
Last wk treatment		2.0 (1.6)	1.7 (1.2)			
1-mth post-treatment		0.6 (1.2)	1.3 (1.2)			
3-mth post-treatment		0.6 (1.2)	0.9 (0.9)			
PG-SGA Box 3 mean (s.d)				β = 0.53	0.41	−0.74, 1.79
First wk treatment		2.6 (2.8)	1.1 (2.2)			
Last wk treatment		4.8 (4.0)	4.7 (3.9)			
1-mth post-treatment		2.8 (4.0)	3.4 (3.7)			
3-mth post-treatment		1.9 (2.9)	2.3 (2.9)			
Unplanned admissions, *n*		6	64	OR = 3.00	0.03	1.13, 8.02
LOS in days, *n*		26	253	β = −1.70	0.34	−1.81, 5.19
RT interruptions, *n* (%)		3 (25)	5 (7)	OR = 0.24	0.09	0.5, 1.25
Reactive NGT, *n* (%)		3 (23)	21 (30)	OR = 0.75	0.65	0.22, 0.26
Mortality at 2-yrs *n* (%)		4(31)	5(7)	β = −1.58	<0.01	−2.27, −0.89

Abbreviations: HPV = Human papillomavirus; CI = confidence interval; IRR = Incidence Rate Ratio; LOS = length of stay; LOW = loss of weight; NGT = Nasogastric Tube; OR = odds ratio; PG-SGA = Patient-Generated Subjective Global Assessment; RT = radiotherapy; s.d = standard deviation.

**Table 4:** HPV-positive and HPV-negative in a linear mixed model of mean Health Related Quality of Life (QLQ C30) across end of treatment, 1 months and 3 months post treatment.

Variable		*β* Statistic	*p*-Value	95% CI
Total HRQOL score *		2.63	0.50	0.26, 0.68
	Global Health *	2.83	0.53	−6.17, 11.83
Functional Outcomes *				
	Role functioning	−2.61	0.70	16.23, 10.99
	Physical Functioning	2.91	0.54	−6.54, 12.37
	Emotional Functioning	0.47	0.91	−7.50, 8.43
	Cognitive Functioning	−14.34	<0.01	−75, −5.04
	Social Functioning	−3.03	0.58	−13.81, 7.77
Symptom Scales #				
	Fatigue	−3.44	0.53	−14.06, 7.18
	Nausea & Vomiting	−5.69	0.33	−17.94, 6.56
	Pain	9.59	0.11	−2.26, 21.44
	Dyspnea	−5.69	0.21	−14.57, 3.20
	Insomnia	0.82	0.89	−11.27, 12.91
	Appetite Loss	−10.91	0.19	−27.33, 5.50
	Constipation	4.28	0.47	−7.42, 15.99
	Diarrhea	−2.99	0.48	−11.29, 5.29
	Financial difficulties	2.48	0.75	−12.73, 17.69

Abbreviations: HPV = Human papillomavirus; CI = confidence interval; HRQOL = Health-Related Quality of Life Questionnaire (QLQ-C30). * Higher score is better. # Lower score is better.

## Data Availability

Not applicable.
